# Supplementary material for: Feature-based learning improves adaptability without compromising precision
Source: Nat Commun. 2017 Nov 24;8:1768. doi: 10.1038/s41467-017-01874-w (PMC5700946; doi:10.1038/s41467-017-01874-w)
Supplement: Supplementary file 1 — Supplementary Information [file 41467_2017_1874_MOESM1_ESM.pdf]

Supplementary Information

Feature-based learning improves adaptability without compromising precision

Shiva Farashahi*, Katherine Rowe*, Zohra Aslami, Daeyeol Lee, Alireza Soltani

Supplementary Figures

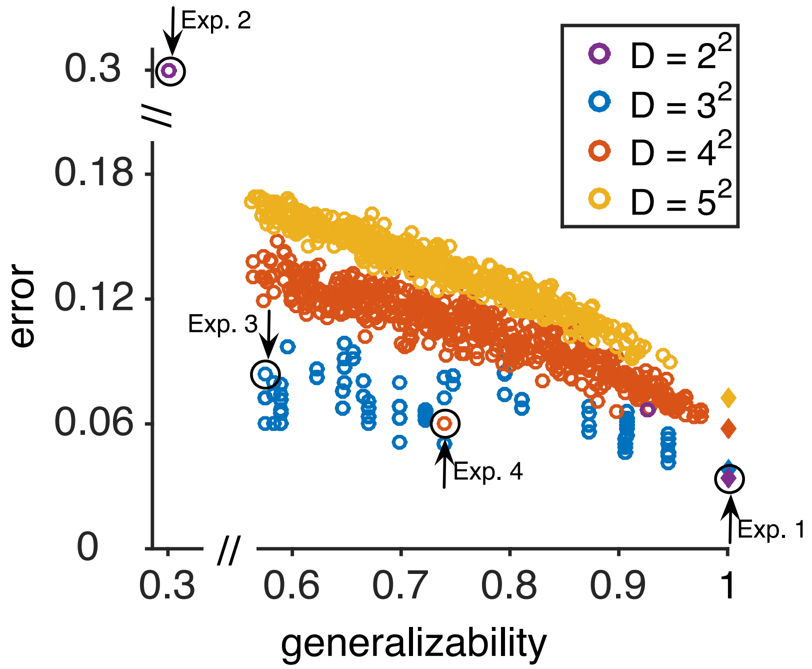

Supplementary Figure 1. Error in the estimation of reward probabilities using the reward values of features. Plotted is the mean of absolute difference between the estimated reward probabilities based on features (Eq. 1) and the actual reward probabilities, as a function of the generalizability index separately for environments with different values of dimensionality. The error increases with smaller generalizability and with larger dimensionality. Error values for fully generalizable environments are plotted with filled diamonds. The black circles indicate error values for Experiments 1 to 4. The generalizability and error for reward matrices used in Experiments 3 and 4 are different from environments with similar dimensionality because of the removal of a few non-informative objects in these experiments. The shown relationship between the estimation error and generalizability predicts that adopting feature-based models is advantageous in more generalizable environments.

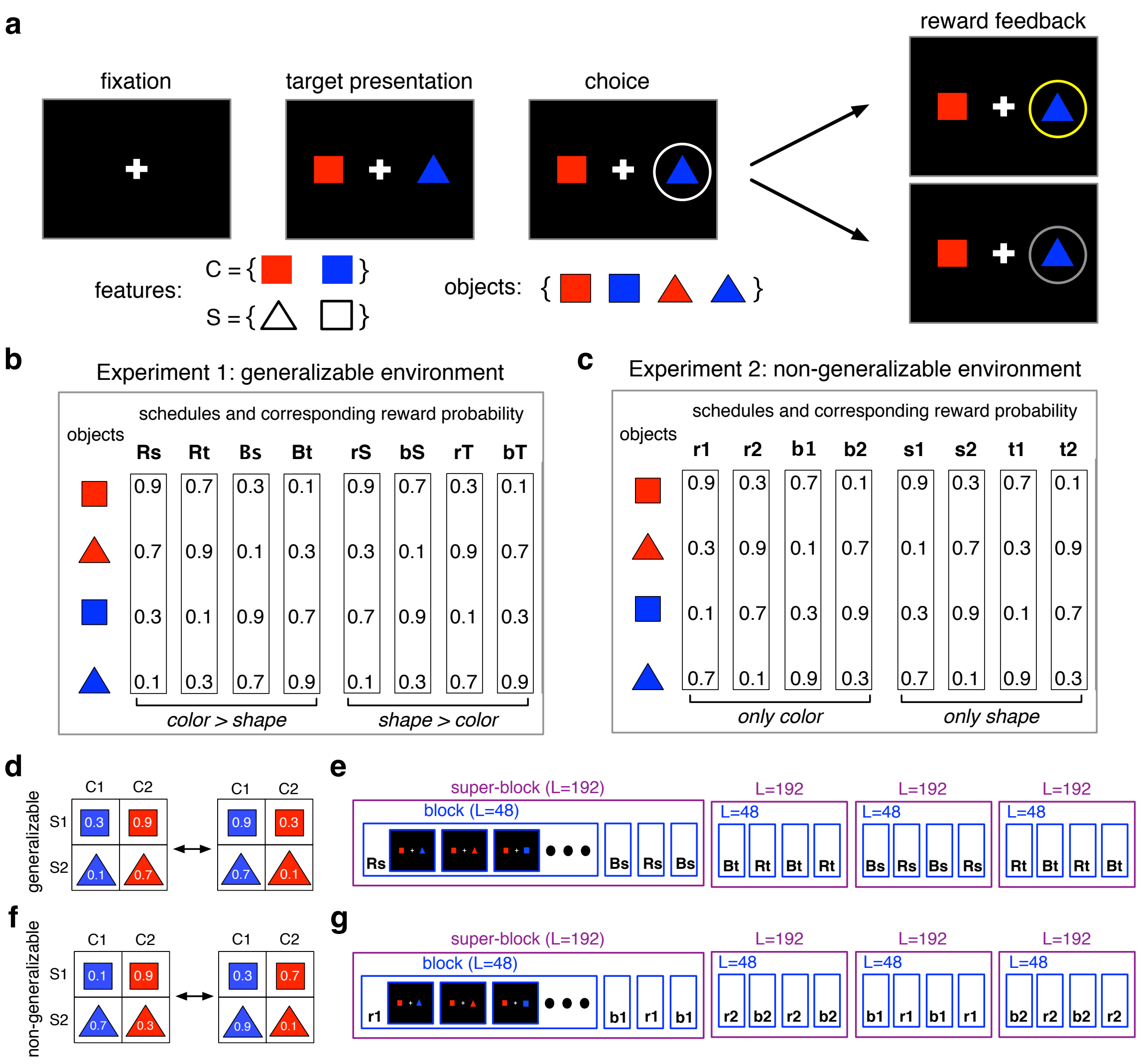

Supplementary Figure 2. Timelines and reward schedules of Experiments 1 and 2. (**a**) In each trial, the subject chose between two objects (colored shapes) and was provided with reward feedback (reward or no reward) on the chosen object. The inset shows the set of all objects used during Experiments 1 and 2. (**b**) Alternative schedules for assigning reward probability to individual objects based on a generalizable rule (Experiment 1). Each column represents a different schedule, and each row next to a given symbol indicates the reward probabilities associated with that symbol in different schedules. Reward schedules are coded to show which feature (color or shape) is more informative and which feature instances are more rewarding. For example, ‘Rs’ indicates that red objects are more rewarding than blue objects, squares are more rewarding than triangles, and color (‘R’) is more informative than shape (‘s’; *color > shape*). (**c**) Alternative schedules for assigning reward probability to individual objects based on a non-generalizable rule (Experiment 2). For these schedules, only one of the two features was on average informative about reward values (e.g. red for ’r1’ schedule; *only color*). (**d-e**) Examples of generalizable

environments constructed by switching between blocks of generalizable reward schedules every 48 trials. (**f-g**) Examples of non-generalizable environments constructed by switching between blocks of or non-generalizable reward schedules every 48 trials.

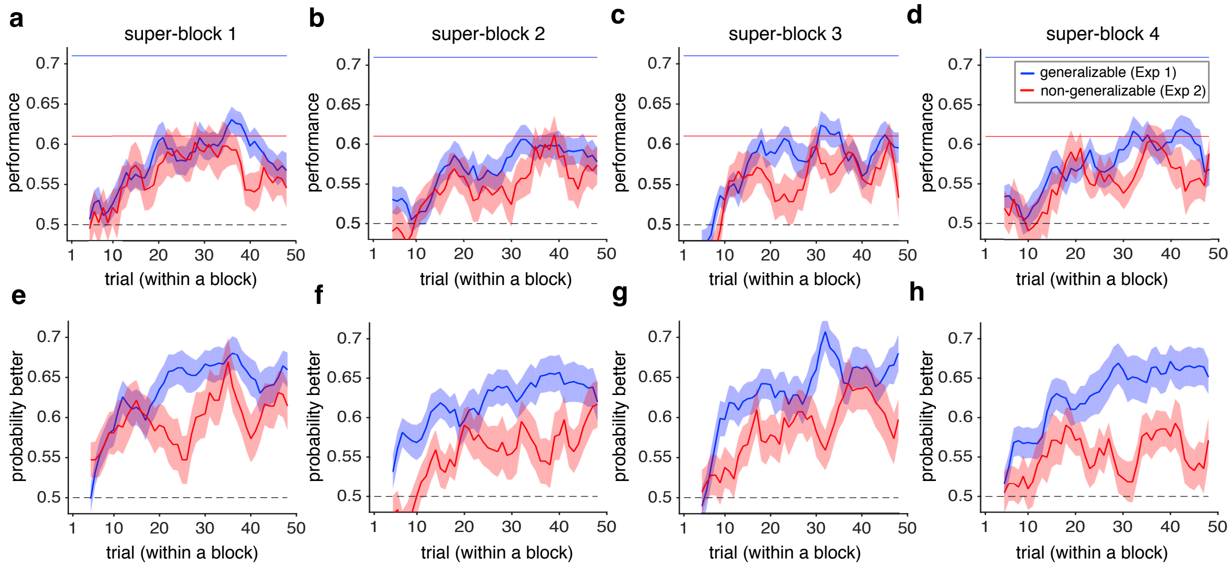

Supplementary Figure 3. Time course of learning during Experiments 1 and 2. (**a-d**). Plotted is the average harvested reward on a given trial during the four super-blocks of Experiments 1 and 2 across all subjects. The shaded areas indicate s.e.m. and the dashed line shows chance performance. The solid blue and red lines show the maximum performance based on the feature-based approach in the generalizable and non-generalizable environments, respectively, assuming that the decision maker selects the more rewarding option based on this approach on every trial. The maximum performance for the object-based approach was similar in the two environments and equal to that of the feature-based approach in the generalizable environment. (**e-h**). Plotted is the average probability of choosing the more rewarding option on each trial during the four super-blocks of Experiments 1 and 2. Overall, performance increased and learning improved over the course of each block and dropped after reversal in both experiments. There was no evidence for different performance in early and late parts of the experiments.

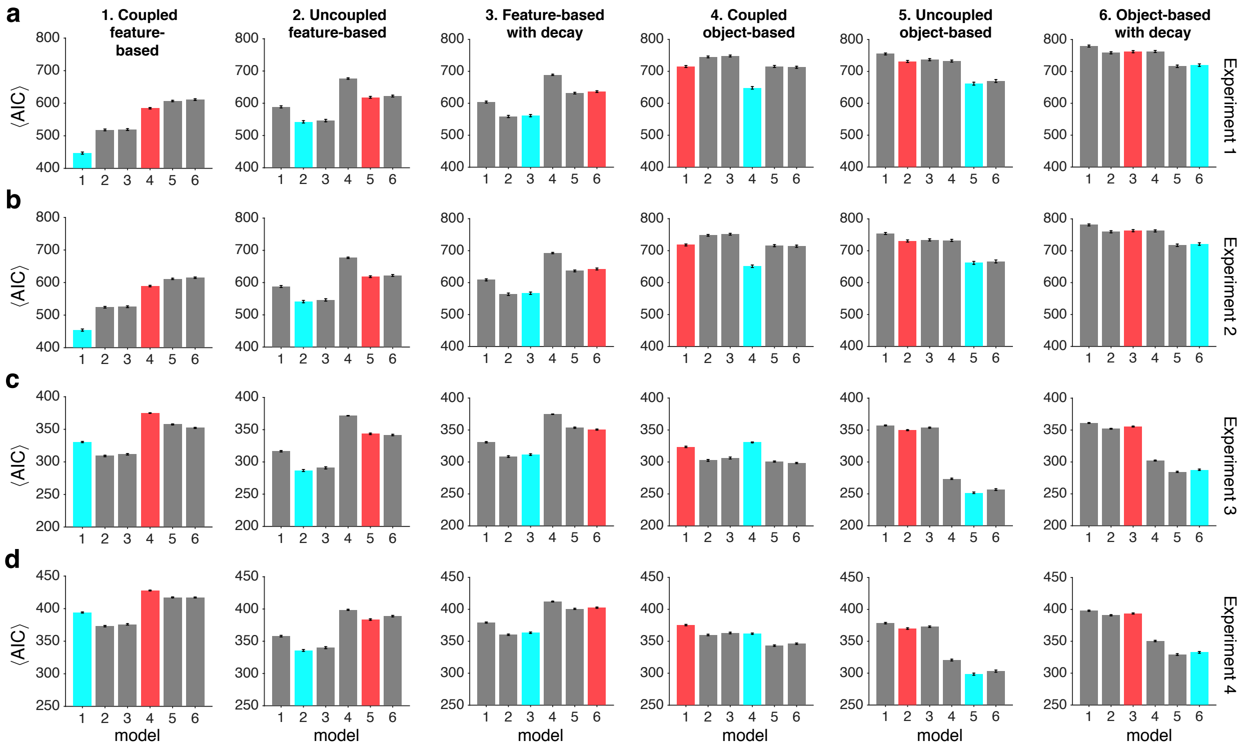

Supplementary Figure 4. Comparison of the goodness-of-fit for the simulated data using various models in Experiments 1 to 4. Each column shows results generated with a given model (numbered 1 to 6) and row **a** to **d** correspond to Experiments 1 to 4, respectively. Plotted is the average AIC (Akaike information criterion) over all sets of parameters (mean ± s.e.m.) for data generated with one of the six models in Experiments 1 to 4 and fit with each of the six models. The results for the model used to generate data in a given experiment and its object-based or feature-based counterpart are highlighted in cyan and orange, respectively. The model used to generate the data provided the best fit with a few exemptions: coupled feature-based or object-based models in Experiment 3 and 4. Even for those models, fits based on the models with a similar learning approach (object-based or feature-based) were better than the corresponding object-based or feature-based models, indicating that the learning approach was identifiable in all cases.

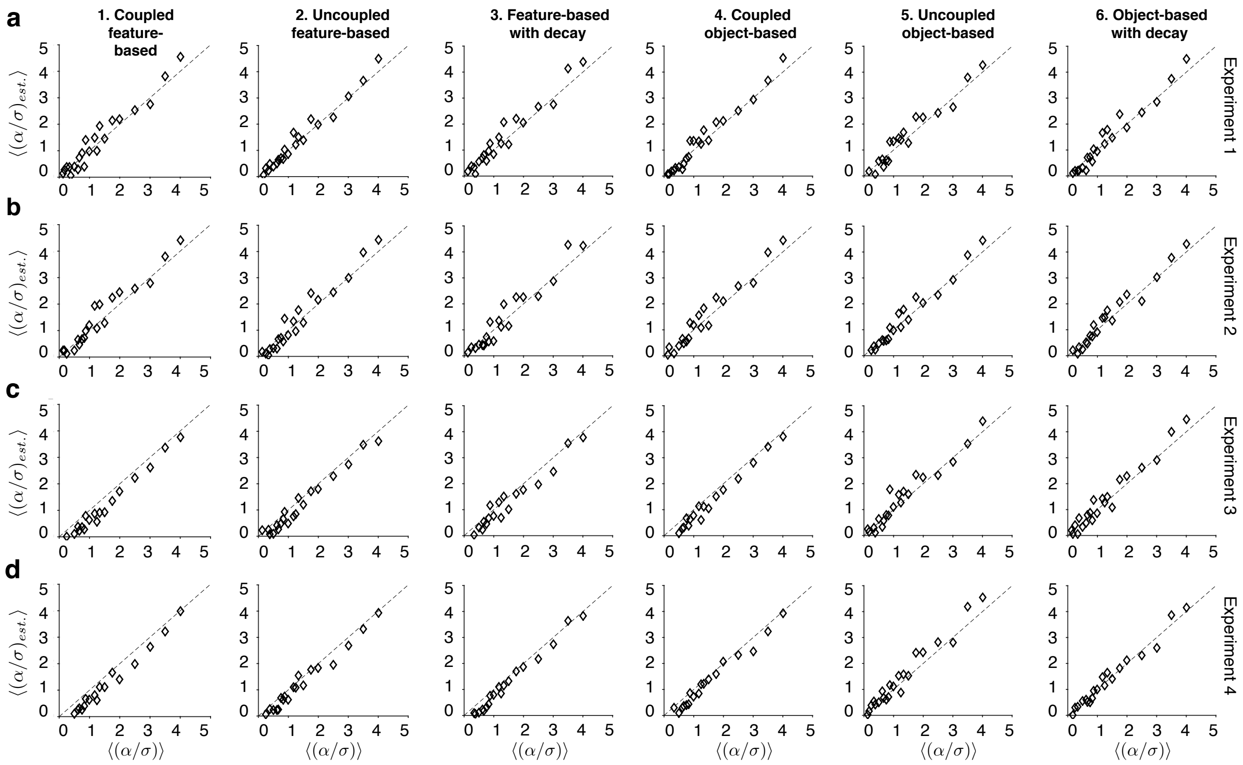

Supplementary Figure 5. Comparison of the estimated versus actual model parameters using the same models used to generate data in Experiments 1 to 4. Each column shows results generated and fit with a given model (numbered 1 to 6) and row **a** to **d** correspond to Experiments 1 to 4, respectively. Plotted is the average of the estimated ratio of the learning rate to the stochasticity in choice versus the actual ratio of the learning rate to the stochasticity, for data generated with one of the six models in Experiments 1 to 4 and fit with the same model. We used the ratio of the learning rate to the stochasticity in choice as the measure because these two parameters influence choice similarly (i.e. a scaled version of the two parameters results in very similar choice behavior). Overall, our fitting procedure allowed accurate estimation of the actual model parameters.

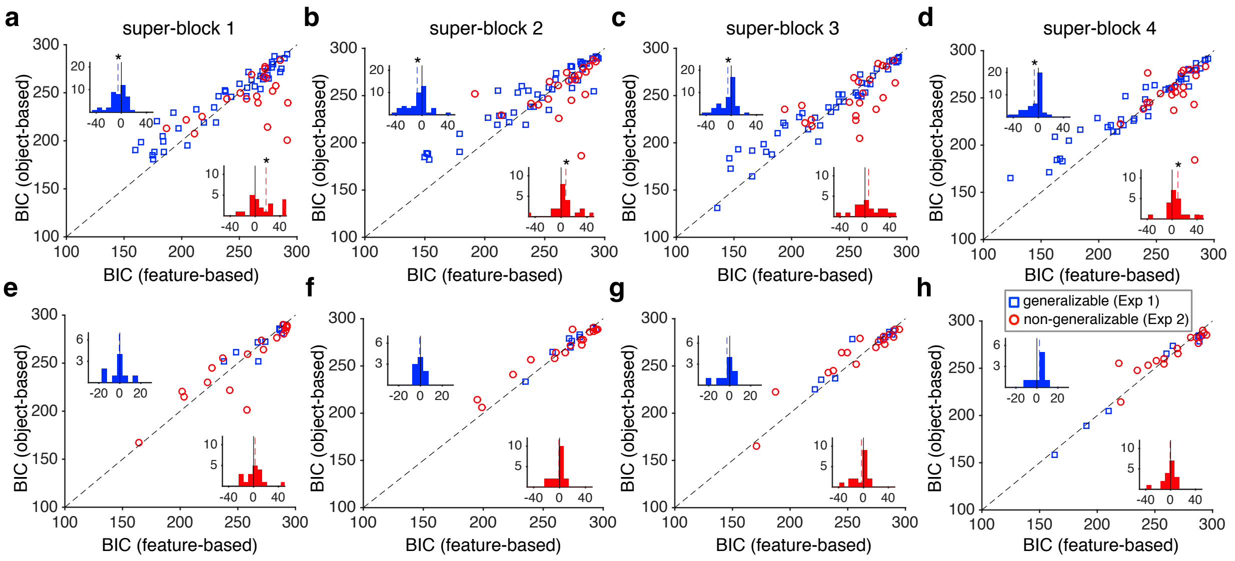

Supplementary Figure 6. (**a-d**) Comparison of the goodness-of-fit based on the best object-based and feature-based model for each individual during each super-block of Experiments 1 and 2. Panels **a** to **d** show the results for super-blocks 1 to 4, respectively. Plotted are the BIC based on the best feature-based and object-based models for each individual and separately for each environment. The insets show histograms of the difference in the BIC from the two best models for the generalizable (blue) and non-generalizable (red) environments. The dashed lines show the medians, and the star shows that the median is significantly different from zero (one-sided sign-rank test, $P < 0.05$). In Experiment 1, the feature-based models provided better fits than object-based models (one-sided sign-rank test; first super-block: P = 0.018, d = 0.39; second super-block: P = 0.0036, d = 0.45; third super-block: P = 0.005, d = 0.50; fourth super-block: P = 0.040, d = 0.46, N = 43). In Experiment 2, the object-based models provided better fits than feature-based models in all super-blocks except the third one (first super-block: P = 0.029, d = 0.50; second super-block: P = 0.038, d = 0.27; third super-block: P = 0.25, d = 0.15; fourth super-block: P = 0.036, d = 0.37, N = 21). Overall, we did not find any evidence for changes in the learning approach during the course of the experiments. These results show that subjects were more likely to adopt the feature-based approach in the generalizable environment and the object-based approach in the non-generalizable environment, and that our results were not driven by two types of behavior during early and late parts of the experiments. (**e-h**) The same as in a-d but for the excluded subjects. Overall, there was no evidence that excluded subjects changed their strategy during the experiments.

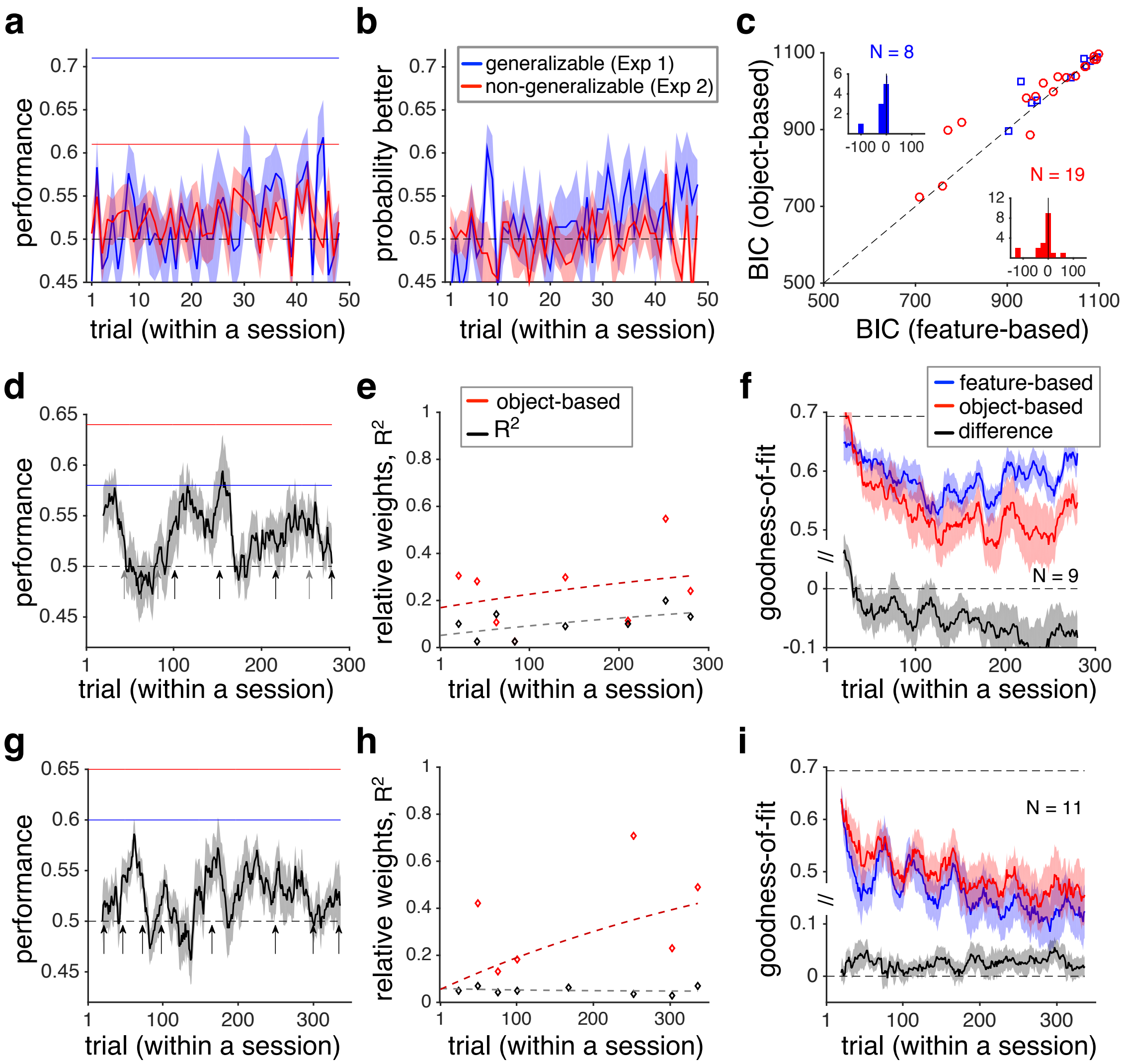

Supplementary Figure 7. Analyses of choice behavior and estimation of the excluded subjects. (**a-b**) Time course of learning during each block of trials in Experiments 1 and 2. Plotted is the average harvested reward (a) or the probability of selecting the better option on a given trial within a block across all excluded subjects. The dashed line shows chance performance. The solid blue and red lines in panel a show the maximum performance based on the feature-based approach in the generalizable and non-generalizable environments, respectively. The maximum performance for the object-based approach was similar in the two environments, and equal to that of the feature-based approach in the generalizable environment. Overall, these subjects failed to learn reward probabilities associated with the four options during Experiment 1 and only exhibited limited learning toward the end of each block in Experiment 2. (**c**) Plotted is the Bayesian information criterion (BIC) based on the best feature-based or object-based models for a given subject, separately for each environment. The insets show histograms of the difference in BIC from the feature-based and object-based models, and the dashed lines show the medians, which is

on top of the zero line. There was no evidence that the excluded subjects adopted a strategy qualitatively different from the one used by other subjects. (**d**) The time course of performance during Experiment 3. Shaded areas indicate s.e.m., and the dashed line shows chance performance. The red and blue solid lines show the maximum performance using the feature-based and object-based approaches, respectively, assuming that the decision maker selects the more rewarding option based on a given approach in every trial. Arrows mark the locations of estimation blocks throughout a session. (**e**) The time course of model adoption measured by fitting subjects' estimates of reward probabilities. Plotted is the relative weight of object-based to the sum of the object-based and feature-based approaches, and explained variance in estimates (R^2) over time. Dotted lines show the fit of data based on an exponential function. (**f**) Transition from feature-based to object-based learning revealed by the average goodness-of-fit over time. Plotted are the average negative log likelihood based on the best feature-based model, best object-based RL model, and the difference between the best object-based and feature-based models during Experiment 3. Shaded areas indicate s.e.m., and the dashed line shows the measure for chance prediction. Overall, excluded subjects moved toward object-based learning over time similarly to what was found for the subjects included in the study. (**g-i**) The same as in **d-f**, but during Experiment 4. Throughout this experiment, feature-based learning provided a better fit for choice behavior of excluded subjects, similarly to that for the subjects included in the study.

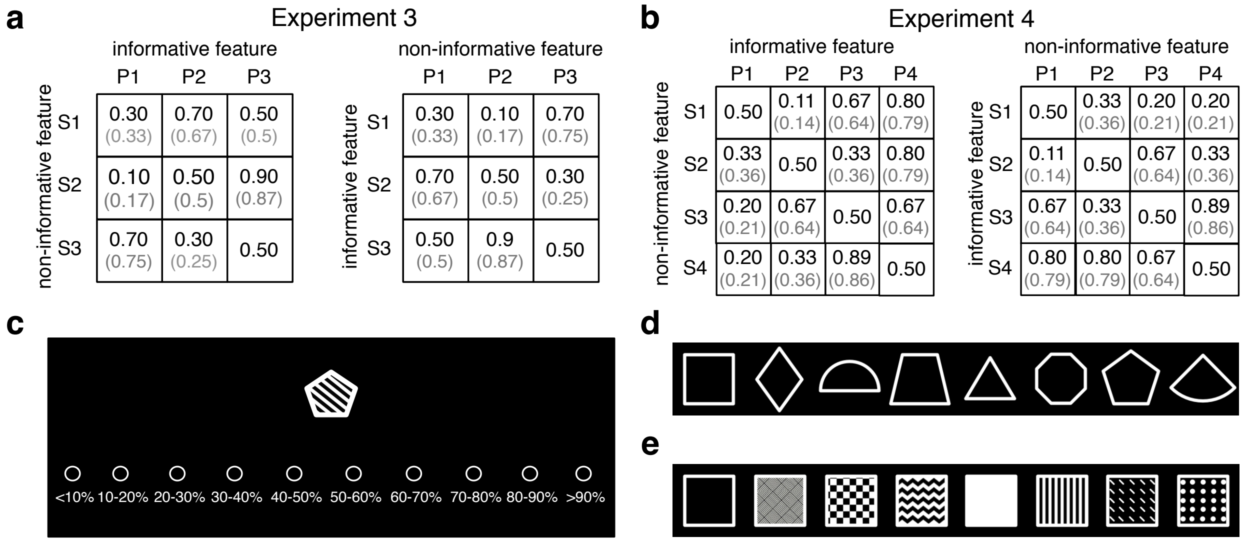

Supplementary Figure 8. Reward probabilities and objects used in Experiments 3 and 4. (**a**) During Experiment 3, reward probabilities were assigned to nine possible objects defined by combinations of two features (S, shape; P, pattern), each of which could take any of three values. Reward probabilities were assigned such that the reward probabilities assigned to all objects could not be determined by combining the reward values of their features (non-generalizable). Numbers in parentheses show the actual probability values used in the experiment due to limited resolution for reward assignment. For the set on the left, the pattern was on average informative about reward (average probability of reward = {0.36, 0.5, 0.63}), whereas shape alone was not informative (average probability of reward = {0.5, 0.5, 0.5}). The opposite was true about the right set. Each subject performed the experiment twice: once when pattern was informative and once when shape was informative, using entirely different sets of shapes and patterns. To shorten the experiment, we excluded object 'S3P3' from the choice set. (**b**). During Experiment 4, reward probabilities were assigned to sixteen possible objects defined by combinations of two features (S, shape; P, pattern), each of which could take any of four values. To shorten the experiment, we excluded objects with reward probability of 0.5 from the choice set. Conventions are the same as in **a**. (**c**) A sample estimation trial during Experiments 3 and 4. On each estimation trial, the subject estimated the probability of reward on an individual object by pressing one of ten keys on the keyboard. (**d**) The set of possible shapes used in Experiments 3 and 4. For each session of the experiment, only three or four (for Experiments 3 or 4, respectively) of these shapes were used for a given subject (randomly chosen). (**e**) The set of possible patterns used in Experiments 3 and 4. For each session of the experiment, only three or four (for Experiments 3 or 4, respectively) of these patterns were used.

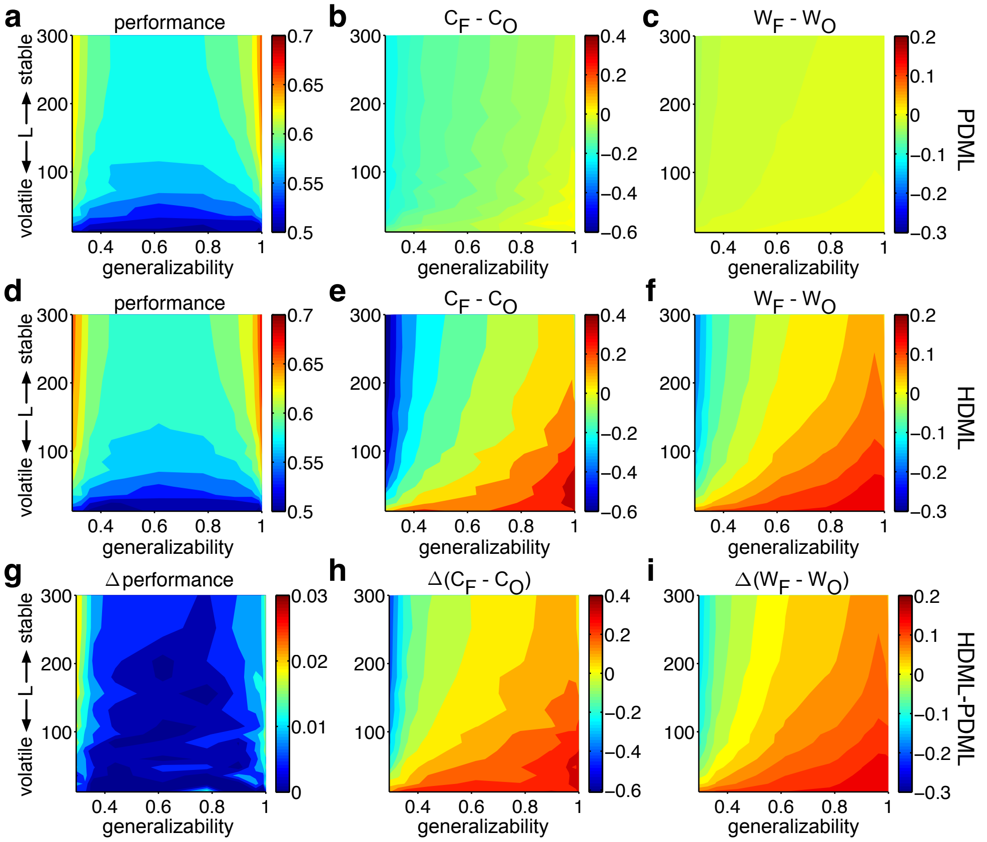

Supplementary Figure 9. The effects of generalizability and volatility (i.e. frequency of changes in reward probabilities) on the models' behavior. (**a**) Performance of the PDML model in various environments with different levels of volatility and generalizability. The color map shows the performance (average harvested reward) for a given value of block length (*L*) and the generalizability index. (**b**) The difference between the strengths of plastic synapses from FVE and OVE neurons onto the final DM circuit (C_F -C_O) in the PDML model. (**c**) The difference between the overall weights of FVE and OVE neurons on the final DM circuit ($W_F - W_O$) in the PDML model. (**d-f**) The same as in **a-c** but for the HDML model. (**g-i**) The difference between the performance, (C_F -C_O), and ($W_F - W_O$) in the HDML and PDML models.

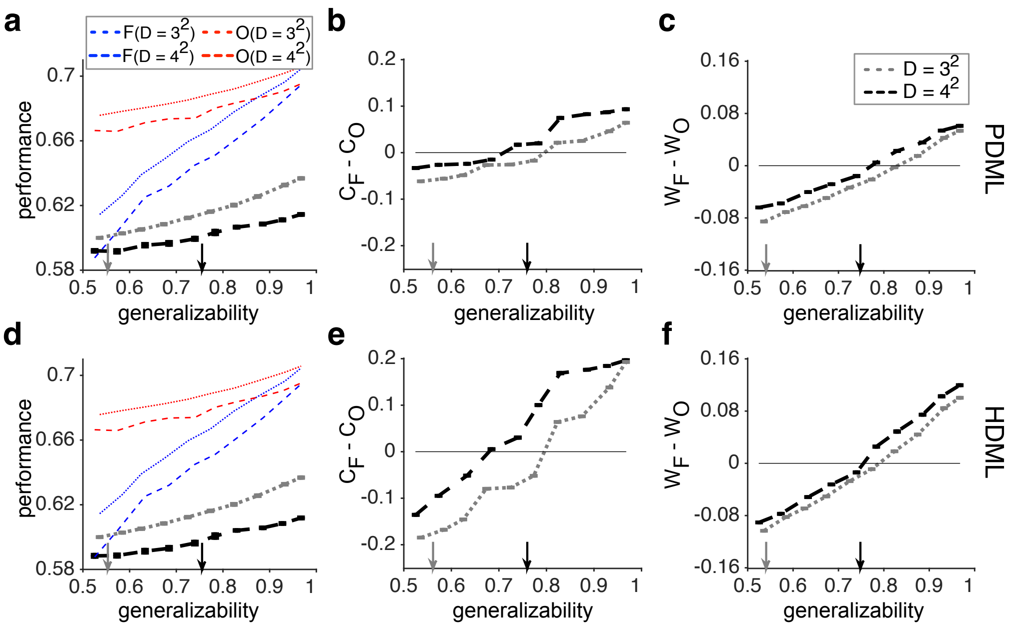

Supplementary Figure 10. The effects of dimensionality and generalizability on the models' behavior. Simulations of the PDML and HDML models are shown in **a-c** and **d-f**, respectively. (**a, d**) Performance of the models as a function of the generalizability index for two environments with nine (gray) and sixteen (black) objects, respectively. The dotted red and blue curves show the maximal performance for object-based (O) and feature-based (F) models, respectively, for $D = 3^2$, as in Experiment 3. The dashed curves show the results for $D = 4^2$, as in Experiment 4. The gray and black arrows indicate the values of the generalizability index used in Experiments 3 and 4, respectively. (**b, e**) The difference between the strengths of plastic synapses from FVE and OVE neurons onto the final DM circuit ($C_F - C_O$) in the PDML model (**b**) and from FVE and OVE neurons onto the signal-selection circuit in the HDML model (**e**). (**c, f**) The difference between the overall weights of FVE and OVE neurons on the final DM circuit ($W_F - W_O$) in the PDML model (**c**), and from FVE and OVE neurons on the signal-selection circuit in the HDML model (**f**).

Model	Coupled feature-based	Uncoupled feature-based	Feature-based with decay	Coupled object-based	Uncoupled object-based	Object-based with decay	
# pars.	5	5	6	4	4	5	
-LL	435.3±11.6***	458.6±8.9***	434.5±11***	451.8±9	474.1±7.4	450.7±8.7	Exp. 1
AIC	880.5±23.1***	927.1±17.8***	880.9±21.9***	911.5±17.9	956.1±14.8	911.4±17.4	
BIC	903.7±23.1*	950.3±17.8***	908.8±21.9**	930.1±17.9	974.7±14.8	934.6±17.4	
-LL	478.2±7.7	493.2±5.3	477.2±7.6	466.4±7.8	494.8±5.5	462.4±7.8*	Exp. 2
AIC	966.5±15.4	996.5±10.6	966.4±15.2	940.8±15.7^{+}	997.6±10.9	934.7±15.6*	
BIC	989.7±15.4	1019.7±10.6	994.3±15.2	959.3±15.7^{+}	1016.1±10.9	957.9±15.6*	
-LL	328.1±4.8	337.0±5.4	323.3±5.6	330.0±6.9	331.6±5.6	290.8±7.9**	Exp. 3
AIC	666.2±9.6	683.9±10.9	658.7±11.2	668.0±13.9	671.2±11.3	591.7±15.7**	
BIC	687.9±9.6	705.6±10.9	684.6±11.2	685.3±13.9	688.5±11.3	613.3±15.7***	
-LL	377.6±6.5***	378.5±6.8***	336.6±9.1**	409.3±4.1	409.6±3.9	350.9±6.8	Exp. 4
AIC	765.2±13.1***	767.1±13.7***	685.2±18.1**	826.6±8.3	827.2±7.8	711.9±13.7	
BIC	787.8±13.1***	789.6±13.7**	712.3±18.1*	844.6±8.3	845.3±7.8	734.4±13.7	

Supplementary Table 1. Comparison of the goodness-of-fit measures in all experiments. Reported are the goodness-of-fit measures, negative log likelihood (-LL), Akaike information criterion (AIC), and Bayesian information criterion (BIC), averaged over all subjects (mean ± s.e.m.) for three feature-based RLs and their object-based counterparts for Experiments 1 to 4. The model providing the best fit in a given experiment and its object-based or feature-based counterpart are highlighted in cyan and orange, respectively. Each feature-based RL was compared with its object-based counterpart using a two-sided, sign-rank test. The significance level of the test is coded as: 0.01 < P < 0.05 (*), 0.001 < P < 0.01 (**), and P < 0.001 (***).

Supplementary Note 1

Ability of HDML and PDML to adopt the proper model of the environment. To further compare the HDML and PDML models, we tested the overall performance and the ability of these models to adopt the feature-based vs. object-based approach in a large set of environments and examined how interactions between generalizability, frequency of changes in reward probabilities (volatility), and dimensionality affect the behavior of these models.

First, we used the two network models to simulate various environments with different levels of generalizability and volatility. These environments were constructed by varying the relationship between the reward value of each object and the reward values of its features, and changing the block length, i.e. the number of trials where reward probabilities were fixed (see Methods). The maximum and minimum levels of generalizability in these simulations correspond to environments used in Experiments 1 and 2, respectively (Supplementary Figure 1). Both models were able to perform the task in various environments with different levels of volatility and generalizability, but the performance of the HDML model was slightly higher in all environments (Δperformance = 0.001±0.0042 (mean±std); two-sided sign-rank test, $P = 0.0023$, $d = 0.23$; Supplementary Fig. 9a, d, g). More importantly, the difference in the strength of connection from FVE and OVE neurons to the next stage of processing ($C_F - C_O$) was more strongly modulated by generalizability and volatility in the HDML compared to the PDML model. This indicated that HDML was better able to adjust the strength of connections from value-encoding neurons (Supplementary Fig. 9b, e, h). As generalizability or volatility increased, connections between FVE neurons and the signal-selection circuit became stronger than connections between OVE neurons and the signal-selection circuit. Therefore, only the HDML model assigned larger weights to feature-based rather than object-based reward values (larger $W_F - W_O$) as the environment became more generalizable or volatile (Supplementary Fig. 9c, f, i). Overall, these results demonstrated that, although both models were able to perform the task, the HDML model exhibited higher performance and stronger adjustment of connections from the value-encoding neurons to the next level of computation. Therefore, HDML was overall more successful in assigning more graded weights to different learning approaches according to reward statistics in the environment.

Second, we examined the interaction between dimensionality reduction and generalizability in adopting a model of the environment by simulating various environments in Experiments 3 and 4 using the two models. Because dimensionality is a discrete number, we considered two different environments with different number of feature instances (three or four) resulting in dimensionality $D = 3^2$ and $D = 4^2$. We also changed the level of generalizability across different environments (see Methods). Consistent with simulation results for Experiments 1 and 2 presented in Supplementary Figure 9, an increase in generalizability caused both models to assign higher weights to feature-based rather than object-based reward values, but this effect was much stronger for the HDML model (larger positive slopes in Supplementary Fig. 10e-f compared with Supplementary Fig. 10b-c). An increase in dimensionality further biased both models to assign more weight to feature-based compared to object-based reward values.

Overall, the simulation results for the two alternative network models illustrate that the HDML model exhibits higher performance and stronger adjustment to task parameters and reward statistics in the environment. These results indicate that hierarchical decision-making and learning might be more advantageous for adopting the model for learning in dynamic, multi-dimensional environments.
